# The thiophene α-terthienylmethanol isolated from *Tagetes minuta* inhibits angiogenesis by targeting protein kinase C isozymes α and β2

**DOI:** 10.3389/fphar.2022.1007790

**Published:** 2022-10-12

**Authors:** María C. Llorens de los Ríos, Priscila A. Lanza, Cecilia L. Barbieri, María L. González, Macarena Funes Chabán, Gastón Soria, D. Mariano A. Vera, María C. Carpinella, Mariana B. Joray

**Affiliations:** ^1^ Fundación para el Progreso de la Medicina, Córdoba, Argentina; ^2^ Department of Chemistry and Biochemistry, QUIAMM–INBIOTEC–CONICET, College of Exact and Natural Sciences, Universidad Nacional de Mar del Plata, Mar del Plata, Argentina; ^3^ Fine Chemical and Natural Products Laboratory, IRNASUS CONICET-UCC, School of Chemistry, Universidad Católica de Córdoba, Córdoba, Argentina; ^4^ CIBICI CONICET and Department of Clinical Biochemistry, Faculty of Chemical Science, Universidad Nacional de Córdoba, Córdoba, Argentina

**Keywords:** angiogenesis inhibitors, *Tagetes minuta*, α-terthienylmethanol, protein kinase C isozymes, molecular docking, molecular dynamics

## Abstract

**Background:** Tumor angiogenesis is considered as a crucial pathologic feature of cancer with a key role in multidrug resistance (MDR). Adverse effects of the currently available drugs and the development of resistance to these remain as the hardest obstacles to defeat.

**Objetive:** This work explores flora from Argentina as a source of new chemical entities with antiangiogenic activity.

**Methods:** Tube formation assay using bovine aortic endothelial cells (BAECs) was the experiment of choice to assess antiangiogenic activity. The effect of the pure compound in cell invasiveness was investigated through the trans-well migration assay. The inhibitory effect of the pure compound on VEGFR-2 and PKC isozymes α and β2 activation was studied by molecular and massive dynamic simulations. Cytotoxicity on peripheral blood mononuclear cells and erythrocyte cells was evaluated by means of MTT and hemolysis assay, respectively. *In silico* prediction of pharmacological properties (ADME) and evaluation of drug-likeness features were performed using the SwissADME online tool.

**Results:** Among the plants screened, *T. minuta*, showed an outstanding effect with an IC_50_ of 33.6 ± 3.4 μg/ml. Bio-guided isolation yielded the terthiophene α-terthienylmethanol as its active metabolite. This compound inhibited VEGF-induced tube formation with an IC_50_ of 2.7 ± 0.4 μM and significantly impaired the invasiveness of bovine aortic endothelial cells (BAECs) as well as of the highly aggressive breast cancer cells, MDA-MB-231, when tested at 10 μM. Direct VEGFR-2 and PKC inhibition were both explored by means of massive molecular dynamics simulations. The results obtained validated the inhibitory effect on protein kinase C (PKC) isozymes α and β2 as the main mechanism underlying its antiangiogenic activity. α-terthienylmethanol showed no evidence of toxicity against peripheral blood mononuclear and erythrocyte cells.

**Conclusion:** These findings support this thiophene as a promising antiangiogenic phytochemical to fight against several types of cancer mainly those with MDR phenotype.

## Introduction

New blood vessel formation or neovascularization comprises vasculogenesis and angiogenesis (Roskoski, 2017). While the former refers to new blood vessel formation from angioblasts and takes place during embryonic development, the latter involves blood vessel formation from the preexisting vasculature (Marmé, 2018). Under physiological conditions, angiogenesis is usually focal and self-limited in time and constitutes an essential mechanism in biological processes such as reproduction, development and the repair of wounds which is strictly regulated by various chemical mediators (Folkman, 2007). Among these, the vascular endothelial growth factor (VEGF)/VEGFR family plays a key role. Physiological VEGF growth factors include VEGF-A/B/C/D and placental growth factor (PlGF), with VEGF-A (commonly designated as VEGF) as one of the most potent angiogenic cytokines (Roskoski, 2017). VEGF binds two receptors tyrosine kinase, VEGF 1 receptor (VEGFR-1) and VEGF 2 receptor (VEGFR-2) (Ferrara et al., 2003). The latter plays a major role in angiogenic VEGF-driven responses (Wang et al., 2020). Among the proteins located downstream this master receptor, PKC isozymes have a pivotal role in controlling cell proliferation, migration, invasion, tumorigenesis, metastasis, epithelial to mesenchymal transition, and angiogenesis (Xu et al., 2008; Simons et al., 2016; Newton, 2018; Llorens et al., 2019). In particular, α and β2 isozymes were found to be consistently activated as a result of VEGR-2 activation by VEGF in endothelial cells (Xia et al., 1996).

Imbalances between pro- and antiangiogenic stimuli may occur, and result in a pathological angiogenesis (Folkman, 2007). Many diseases, such as psoriasis, rheumatoid arthritis, atherosclerosis, infantile hemangioma and, mainly cancer are linked to increased neovascularization (Folkman, 2007; Haibe et al., 2020). Angiogenesis is an essential process for the growth and progression of cancer cells having a crucial role in metastasis (Hussen et al., 2022). This process is also implicated in the development of MDR by inducing the expression of drug efflux transporters such as MRP1 and MDR-associated genes (Li et al., 2013; Yao and Zhang, 2019).

Although much progress has been made in the field of antiangiogenic therapy, the regulation of this process remains a challenge (Gacche and Meshram, 2014). Most of the currently available treatments comprise large molecules that target VEGF and have been associated with adverse effects and the development of resistance to these (Schwikkard et al., 2019). Even though small molecule-based antiangiogenic drugs that target VEGFR-2 such as axitinib (Inlyta^®^, Pfizer) and sorafenib (Nexavar^®^, Bayer Onyx), have been approved for oncological treatment, intrinsic or acquired resistance and clinical toxicities have also been reported (Huang et al., 2020). Interestingly, there is no small molecule approved by the FDA for the treatment of ocular neovascularization (Schwikkard et al., 2019). While ruboxistaurin (LY333531), a selective inhibitor of protein kinase C β (PKC-β), showed encouraging results in the reduction of visual loss due to diabetic retinopathy (Mansour et al., 2020), after several phase III clinical trials, the overall benefit seemed to be small and this drug remains in investigational status (Deissler and Lang, 2016). These situation urges the development and the improvement of angiogenesis inhibitors (Huang et al., 2020).

In this regard, natural products continue to play a significant role in drug discovery (Funes Chabán et al., 2019a; Crespo et al., 2019). The effectiveness and the structural diversity of plant-derived metabolites are driving their emergence as an important source of bioactive chemical entities (Joray et al., 2017; Funes Chabán et al., 2019b).

This work evaluated the antiangiogenic activity of 40 extracts obtained from plants of Argentina. The plants studied are commonly consumed, mainly as traditional medicines (Goleniowski et al., 2006; Funes Chabán et al., 2019b), which makes them an interesting resource for obtaining effective, low- or non-toxic phytochemicals with antiangiogenic effect (Seukep et al., 2020). Among the active extracts, *T. minuta* L. was the most effective. Bioassay-guided fractionation of *T. minuta* ethanol extract was performed to isolate and identify its active principle. Mechanistic, functional and toxicity assays were conducted. The findings encouraged us to study at a deeper molecular level the probable targets involved. The present study includes a detailed description of its inhibition mechanism.

## Materials and methods

### Chemicals and materials

3-(4,5-dimethylthiazol-2-yl)-2,5-diphenyltetrazolium bromide (MTT), endothelial cell growth supplement, trypsin-EDTA solution and bovine serum albumin (BSA) were purchased from Sigma-Aldrich Corporation (St Louis, MO, United States). Axitinib > 99% was purchased from Cell Signaling Technology (Danvers, MA, United States). Suramin sodium was purchased from Santa Cruz Biotechnology (Dallas, TX, United States). Sterile plastic material was obtained from Greiner Bio-One (Frickenhausen, Germany). Dulbecco’s modification of Eagle’s medium (DMEM), fetal bovine serum (FBS) and extracellular matrix (ECM, Matrigel^®^) were purchased from Corning Incorporated (Glendale, AZ, United States). Penicillin, streptomycin and Hema 3 staining kit were purchased from Invitrogen Life Technologies (Carlsbad, CA, United States).

Tube formation assay images were obtained with an Olympus CKX41 inverted microscope. Invasion assay images were captured with an optical microscope (NIKON Eclipse TE2000-U and Digital Sight DS-U1). Boyden chamber and Matrigel-coated polycarbonate membranes (8-μm pore diameter) (NeuroProbe) were used for invasion assays. The stock solutions of the different plant extracts, isolated compounds, and the positive controls, axitinib and suramin sodium, were prepared in ethanol.

Analytical TLC was carried out using silica gel 60 F254 plates from Merck. Silica gel (70–230 mesh, 60 Å) and Sephadex LH20 used for column chromatography were acquired from Sigma-Aldrich. NMR spectra were run in a Bruker AVANCE II 400 spectrometer (Bruker Corporation). HPLC was carried out on a Shimadzu LC-10 AS (Shimadzu Corp., Tokyo, Japan), equipped with a Phenomenex Prodigy 5 μ ODS (4.6 mm × 250 mm) reverse phase column.

### Plant material and extract preparation

The aerial parts of the different plant species were collected in the hills of Córdoba Province, Argentina (between −30.773428 to −31.797760 latitude and −64.109384 to −64.546803 longitude), from November to March. The botanist, Gustavo Ruiz, identified each specimen. Authenticated vouchers were deposited in the “Marcelino Sayago” Herbarium of the School of Agricultural Science, Catholic University of Córdoba.

The plant material was air-dried, powdered and extracted by 48 h maceration with ethanol. After solvent removal, the yield of each extract was calculated as the percentage weight of dried and crushed vegetable matter (see Supplementary Table S1).

### Cell lines and culture conditions

Bovine aortic endothelial cells (BAEC) and the triple-negative human mammary carcinoma cell line, MDA-MB-231, were grown in DMEM supplemented with 20% and 10% heat-inactivated fetal bovine serum (FBS), respectively, and penicillin (100 units/ml)-streptomycin (100 μg/ml). The BAEC media contained 0.03 mg/ml endothelial cell growth supplement. Cell cultures were kept at 37°C in a 5% CO_2_ humidified environment. Cells were sub-cultured twice a week and used when under 20th passage from frozen stocks.

### Bioguided fractionation and isolation of the active principle

The ethanol extract of *T. minuta* (5 g) was initially subjected to vacuum liquid chromatography on silica gel (320 g, 70**–**230 mesh, 11.0 cm × 24.0 cm) using an increasing gradient of hexane-ethyl ether-methanol. From this, 16 eluates were collected and grouped according to their chemical profile in thin-layer chromatography (TLC) in 4 fractions (F1 to F4). In all the steps of the isolation process, the chromatographic fractions were tested in the tube formation experiment at a final concentration of 100 μg/ml. Only fraction F4 resulted active, showing complete inhibition of tube formation. Therefore, it was reprocessed by column chromatography in Sephadex LH20. For this, 0.5 g of sample was dissolved in the mobile phase (hexane-chloroform-methanol 2:1:1) and seeded in the column. The eluates were collected in 36 test tubes and then combined according to the TLC profile in 6 fractions, F4.1 to F4.6. Of these, only fraction F4.5 totally blocked tube formation and was subjected to an additional Sephadex LH20 column chromatography using the previously mentioned mobile phase. This process furnished five fractions designated as F4.5.1 to F4.5.5. The chemical profile in TLC run with hexane-ethyl acetate 80:20 showed that each fraction F4.5.2, F4.5.3 and F4.5.4, contained a single major spot with equal *rf* value ( ≈ 0.29) which reacted differently when revealed with vainillin/sulphuric acid reagent (blue, violet and greenish gray, respectively). From F4.5.3, compound **2** (3.4 mg) was obtained with 90% purity as determined by HPLC. Fractions F4.5.2 and F4.5.4 required further purification. These were independently reprocessed by means of flash reversed-phase chromatography using Biotage SNAP-C18-HS cartridges (12 g silica) and a mobile phase of acetonitrile**-**water 50:50 (flow rate 12 ml/min). This process yielded 1 mg of compound **1** and 2 mg of compound **3** with 96% and 99% purity, respectively. The chemical structures were elucidated by means of 1D and 2D NMR and by comparison of their spectral data with previously reported values (Eachern et al., 1988; Ahmad and Alam, 1996; Ibrahim et al., 2015). Compounds **1** and **2** were respectively identified as the bithiophenes 5′-methyl-[5-(4-hydroxy-1-butynyl)]-2,2′-bithiophene and 5-(4-hydroxybut-1-ynyl)-2,2′-bithiophene, while compound **3** was identified as the terthiophene 5-hydroxymethyl-2,2':5′,2″-terthiophene, also known as α-terthienylmethanol (Figure 2).

### Tube formation assay

The experiment was performed according to Arnaoutova and Kleinman (Arnaoutova and Kleinman, 2010). Briefly, BAECs (1.5 × 10^4^ cells) were placed on a 96-well plate previously coated with Matrigel^®^. The plates were incubated for 18 h (37°C, 5% CO_2_) in the presence and absence of the tested extracts or the pure compounds, at a maximum concentration of 100 μg/ml and 30 μM, respectively, with the addition of VEGF (10 ng/ml) as angiogenic stimuli. Suramin sodium 30 μM or axitinib 10 μM were used as positive inhibition controls. Controls without or with ethanol (negative control) were simultaneously run. The final concentration of vehicle per well in this and all the experiments described herein was 1% v/v. No adverse effects were observed at this concentration. The images were obtained with an inverted microscope and analyzed with the ImageJ software. The tubular structures were quantified. The percentages of inhibition (I%) were calculated as I% = [1−(total tube length treatment/total tube length control)] × 100. Each condition was assessed in triplicate. Each experiment was performed independently three times.

### Cell proliferation assay

The MTT colorimetric assay was performed as previously described (Joray et al., 2015, 2017; González et al., 2018; García Manzano et al., 2020). Briefly, 2 × 10^4^ cells suspended in 100 μl of complete growth medium were seeded in 96-well plates and 24 h-incubated to allow cell attachment. After that, 100 μl of medium were incorporated, containing different concentrations of the antiangiogenic extracts or of the pure compound **3** previously dissolved in ethanol. After 24 h, 20 μl of 5 mg/ml solution of MTT in sterile PBS were added and the plates were incubated for another 4 h. The plates were then centrifuged for 10 min at 2,000 rpm and the supernatants were removed. Finally, the purple formazan crystals were solubilized with 100 μl of DMSO and the absorbance was measured at 595 nm. Ethanol (vehicle, final concentration 1% v/v) was used as a negative control. The percentage of cytotoxicity was calculated as follows: Cytotoxicity (%) = [1− (Optical density of treated cells−Optical density DMSO)/(Optical density of control cells−Optical density DMSO)] × 100.

### Invasion assay

The experiment was performed according to Llorens et al. (2019). Briefly, MDA-MB-231 or BAEC cells (2.5 × 10^4^ cells/well) were seeded in 0.1% BSA/DMEM in the upper compartment of a Boyden chamber (NeuroProbe) and the cells were treated with compound **3** (30 and 10 μM) or axitinib (0.1 μM) as a positive control (Li et al., 2011; Chiew et al., 2017). Matrigel^®^-coated polycarbonate membranes (8-μm pore diameter) were used to separate the upper and the lower compartments. In the lower chamber, DMEM medium containing 10% FBS for MDA-MB-231 cells or 10 ng/ml VEGF for BAEC cell line were used as chemoattractants. After an incubation period of 16 or 24 h at 37°C, 5% CO_2_, for MDA-MB-231 and BAEC, respectively, invasive cells in each well were counted in five random fields by contrast microscopy using an optical microscope and the ImageJ/Fiji software. Each condition was assessed in triplicate in three independent experiments.

### Molecular modeling simulations

The X-ray crystallographic structure of VEGFR-2 co-crystallized with sorafenib as inhibitor (PDB: 4ASD) (McTigue et al., 2012) was used as starting structure. Incomplete loops were modeled using the structures of VEGFR-2 kinase domain in complex with a nicotinamide inhibitor and the crystal structure of VEGFR-2 (juxtamembrane and kinase domains) in complex with axitinib (PDB 2P2I and 4AGC, respectively) (Hodous et al., 2007; McTigue et al., 2012), and optimized with the AMBER tools (details below). The structures of the small molecules sorafenib, axitinib, the compounds in Figure 9, and the subject compound, in their protonation states at physiological pH were subjected to a conformational search in relevant cases at the semiempirical PM6 level of theory, and then fully optimized using quantum calculations within the DFT framework at the PBE0/6-31G(d,p) level of theory. The minimum nature in each case was characterized by diagonalization of the Hessian matrix using the Gaussian 16 Rev. A03 program (http://www.gaussian.com) (Frisch, 2016). The genetic docking algorithm was Lamarckian as implemented in Autodock4.2.6, using a population of 150 individuals and 2,000 runs per ligand, with termination criteria for each of the runs of 100,000 generations or 6,000,000 energy calculations, the rest of the options being left to the program default (Morris et al., 2009).

In order to validate the protocol, a blind docking was performed in the case of sorafenib (co-crystallized), using a big box including two thirds of the structure, including potential pockets apart from the so-called HYDI (where the sorafenib actually was trapped in the X-rays) and the allosteric HYD II (mainly involving Leu889, Ile892, Val898 and Ile1044) (Modi and Kulkarni, 2019). The protocol was found to yield an excellent (RMSD of 0.86 Å) prediction of the crystallographic pose (See Supplementary Figure S1 for the superposition of the experimental and the lowest energy docked poses). Similar checks were made in the case of the PKC isozymes α (PDB 3IW4) and β (PDB 2I0E) with the co-crystallized NVP and PDS inhibitors, respectively (Neil Grodsky et al., 2006; Wagner et al., 2009).

The most stable docked structure for each protein/inhibitor complex was used as starting geometry for the molecular dynamics (MD) simulations. For the cases of compound **3** and sorafenib with VEGFR-2, a secondary docked pose in the allosteric hydrophobic pocket (roughly HYD-II) was also simulated. The protein/ligand complexes were prepared using the AMBER18 leap, parmchk2 and antechamber facilities for the parametrization of the non-protein residues (i.e., the inhibitors) (Wang et al., 2006). The charges were obtained with the -bcc option, calling the internal AM1 of the sqm module of AMBER (Wang et al., 2006). The general setup for the MD simulations was as follows: I) 250 steepest descent minimization steps of the whole system, keeping the protein tightly restrained and embedded into a box of TIP3P water molecules with a minimum distance of 10 Å to each wall, and Cl^−^ and Na^+^ counter-ions to reach electro-neutrality as required. II) 6500 conjugate gradient minimization steps of the whole system. III) 100 ps slowly heating in the NTV ensemble with the protein positionally restrained in the backbone. IV) 85–100 ns of simulation in the NTP ensemble, at 1 atm and 300 K. Procedures III-IV were repeated in two or three independent trajectories using the Andersen thermostat and barostat (Andersen, 2008). In the case of the VEGFR-2, additional simulations were performed by applying a 25.0 kcal/Å^2^ harmonic restraint for the backbone atoms of the residues known to be located in the transmembrane region. Since no sensitive differences were observed with respect to the fully unrestricted trajectories, those details will not be further discussed. Electrostatic interactions were computed using the Particle Mesh Ewald (PME) method with a cutoff of 10 Å (Essmann et al., 1998). Bonds involving hydrogen atoms were constrained using the SHAKE algorithm, allowing for an integration time step of 0.002 ps. The integration was made using the pmemd. CUDA module of the AMBER18 program (Case et al., 2021) with the auxiliary force field GAFF for the ligands and ff14SB for the protein (Maier et al., 2015). The trajectories were analyzed using standard AMBER cpptraj analysis tools. The free energy calculations were made using the mmpbsa module of AMBER 18 by applying Poisson-Boltzman (PB) and Generalized Born (GB) models (Genheden and Ryde, 2015). The energy analyses were made for the last 10 ns of simulation as the average over at least two independent trajectories. The frames were sampled once each 5–10 saved frames (saving 1 frame every 10 ps, thus ensuring that the energy self-correlation is small enough). The clustering analyses for visualization of representative conformations and rendering of some figures were made using Chimera 1.15 (Pettersen et al., 2004). Most graphic rendering was prepared using Chimera and VMD 1.9.3 (Humphrey et al., 1996).

### Cytotoxicity on peripheral blood mononuclear cells and hemolysis assay

In order to evaluate toxicity in peripheral blood mononuclear cells (PBMC), increasing concentrations of compound **3** from 2.5 to 100 μM were tested by MTT assay, as previously reported (Joray et al., 2015, 2017; García Manzano et al., 2020; Laiolo et al., 2021a) with an incubation period of 48 h. The hemolysis assay was performed with the compound at a maximum concentration of 100 μM (Joray et al., 2011; Funes Chabán et al., 2019a; Laiolo et al., 2021a, 2021b). For both experiments, fresh heparinized blood was collected from healthy human volunteer donors not receiving any treatment. Ethical approval was obtained from the Catholic University of Córdoba Research Ethics Board and donors signed informed consents.

### Pharmacological properties and drug-likeness estimation for compound 3

*In silico* prediction of pharmacological properties (ADME) and evaluation of drug-likeness features of compound **3** were performed using the SwissADME online tool that is freely available at http://www.swissadme.ch. Pharmacokinetic parameters including blood-brain barrier permeation, gastrointestinal absorption and cytochrome P450 binding were estimated. Drug-likeness was assessed with the filters Lipinski, Ghose, Veber, Egan, and Muegge, and its bioavailability score was predicted. The presence of problematic substructures, such as Pan Assay Interference Structures (PAINS) and Structural Alerts (Brenk alerts), together with its synthetic accessibility, were also determined. Details about the SwissADME web service and its underlying methodologies can be consulted in Daina et al. (2017).

### Statistical analyses

The results are expressed as mean ± SE. Data were analyzed using ANOVA followed by Dunnett’s post-hoc test. GraphPad Prism 7.0 was used, with *p*-values ≤ 0.05 defined as statistically significant. Each experiment was repeated at least three times.

## Results

### The ethanol extracts from *Bidens pilosa* and *T. minuta* showed remarkable antiangiogenic effect

The antiangiogenic activity of 40 ethanol extracts obtained from plants mostly native to Argentina was evaluated *in vitro* by the tube formation assay, which is one of the most specific and preferred tests for assessing angiogenesis *in vitro* (Arnaoutova and Kleinman, 2010; Sanz-Nogués and O’Brien, 2016). The inhibitory values determined for the tested extracts can be found in Supplementary Table S1.

**FIGURE 1:**
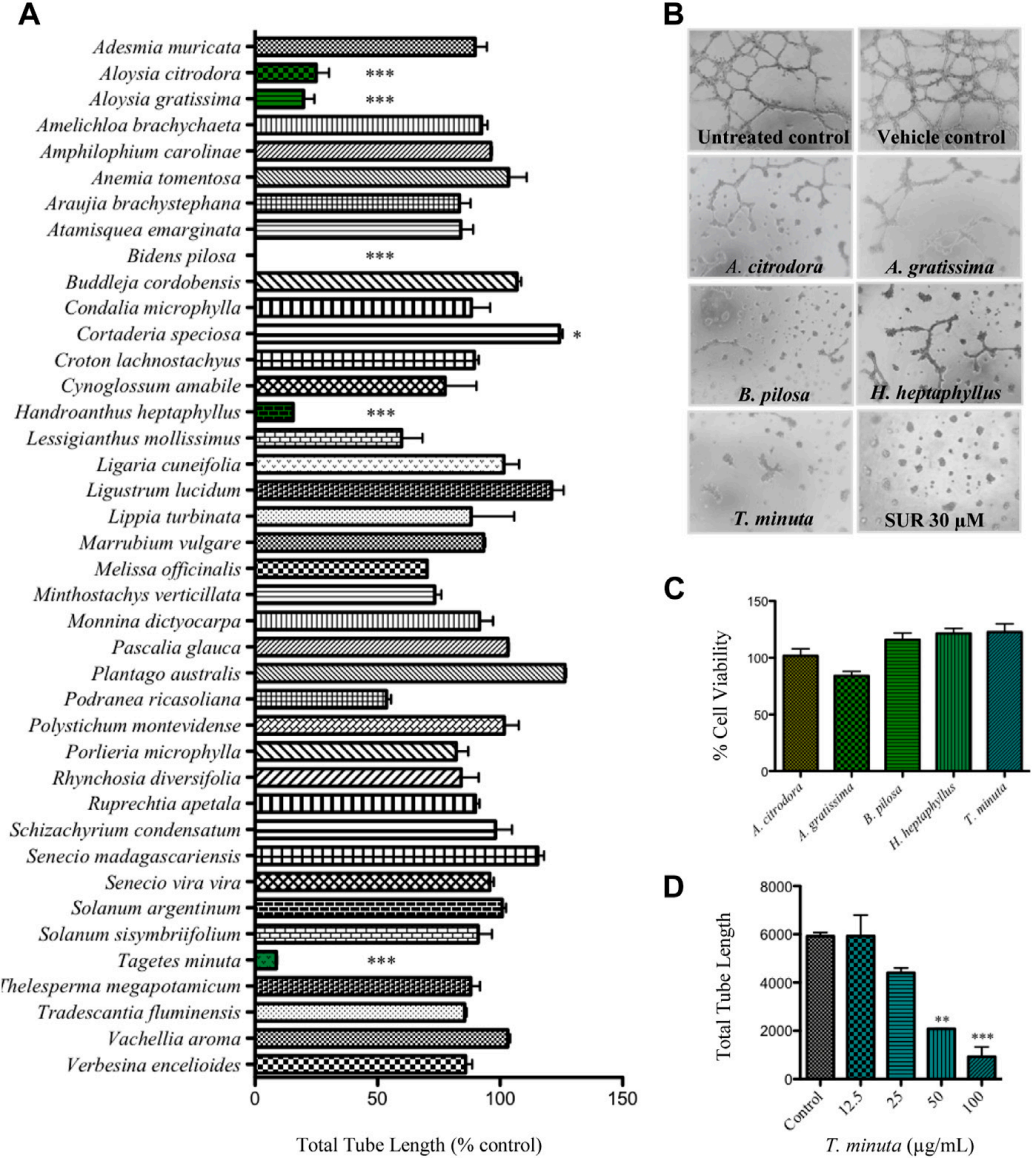
The antiangiogenic activity of the plant ethanol extracts was assessed by means of the tube formation assay. **(A)** After 18 h incubation, the BAEC endothelial tubular network was analyzed, and the total tube length was quantified using the ImageJ software. Data are shown as percentage of the respective control (1% ethanol, vehicle) and represented as the mean ± SEM. Statistical analysis was performed by ANOVA followed by Dunnett’s post-hoc test (***p < 0.001, *p < 0.05). **(B)** Representative images of BAEC cell capillary-like structures when treated with 100 μg/ml of the antiangiogenic plant extracts, untreated control, negative control (vehicle) and the suramin sodium positive control (SUR, 30 μM). **(C)** Cell viability after 24 h treatment with the antiangiogenic extracts was assessed in terms of the MTT assay. The percentages of cell viability referred to the negative control (1% ethanol, vehicle) are expressed as the mean ± SEM. **(D)** Dose-response curve of *Tagetes minuta* extract. The bars show the total tube length expressed as the mean ± SEM. Statistical analysis was performed by ANOVA followed by Dunnett’s post-hoc test (***p < 0.001, **p < 0.01).

The extracts obtained from *Aloysia citriodora, Aloysia gratissima, B. pilosa, Handroanthus heptaphyllus*, and *T. minuta* significantly inhibited tube formation at 100 μg/ml with tube length values lower than 25% compared to control (Figure 1A). No differences between untreated and negative control (1% ethanol, vehicle) were observed.

Representative images of the treatments with these antiangiogenic extracts and the respective controls are shown in Figure 1B. *B. pilosa* and *T. minuta* extracts were the most effective, with tube formation inhibitory values of 100 and 95.5 ± 4.5%, respectively. In order to rule out the possibility that a direct toxic effect could be implicated in the activity observed, the cytotoxicity of the antiangiogenic extracts at 100 μg/ml toward the BAEC cells was evaluated by means of MTT assay. As shown in Figure 1C, after 24 h of treatment, the cell viabilities were 100% for all the treatments except for the ethanol extract of *A. gratissima*, which showed a cell viability of 84.0 ± 3.9%. The extract of *T. minuta* was selected to be submitted to the bioassay-guided isolation of its antiangiogenic principle. With the aim of determining the IC_50_ value of *T. minuta*, serial two-fold dilutions of this extract were tested by means of the tube formation assay (Figure 1D) throwing a calculated IC_50_ of 33.6 ± 3.4 μg/ml.

### An antiangiogenic terthiophene was isolated from *T. minuta*

**FIGURE 2:**
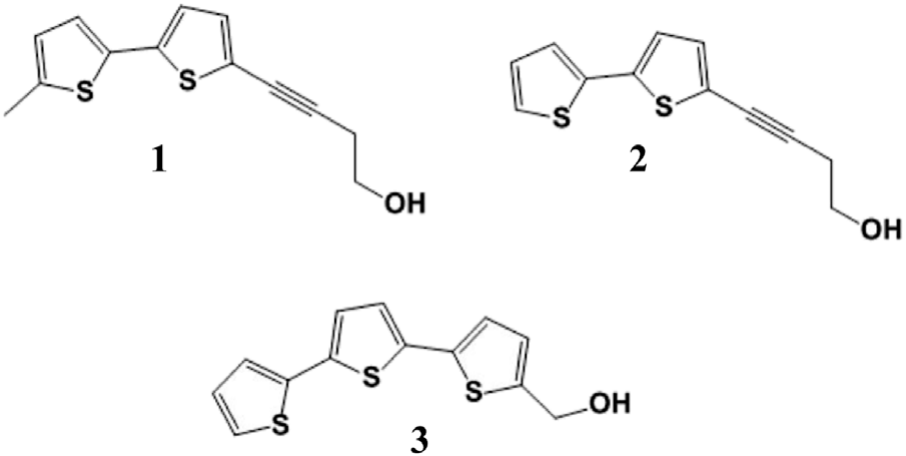
Chemical structures of 5′-methyl-[5-(4-hydroxy-1-butynyl)]-2,2′-bithiophene (1), 5-(4-hydroxybut-1-ynyl)-2,2′-bithiophene (2) and α-terthienylmethanol (3).

Bioactivity-guided fractionation of *T. minuta* ethanol extract was successfully performed. From this process, compounds **1**, **2** and **3** were isolated (Figure 2).

**FIGURE 3:**
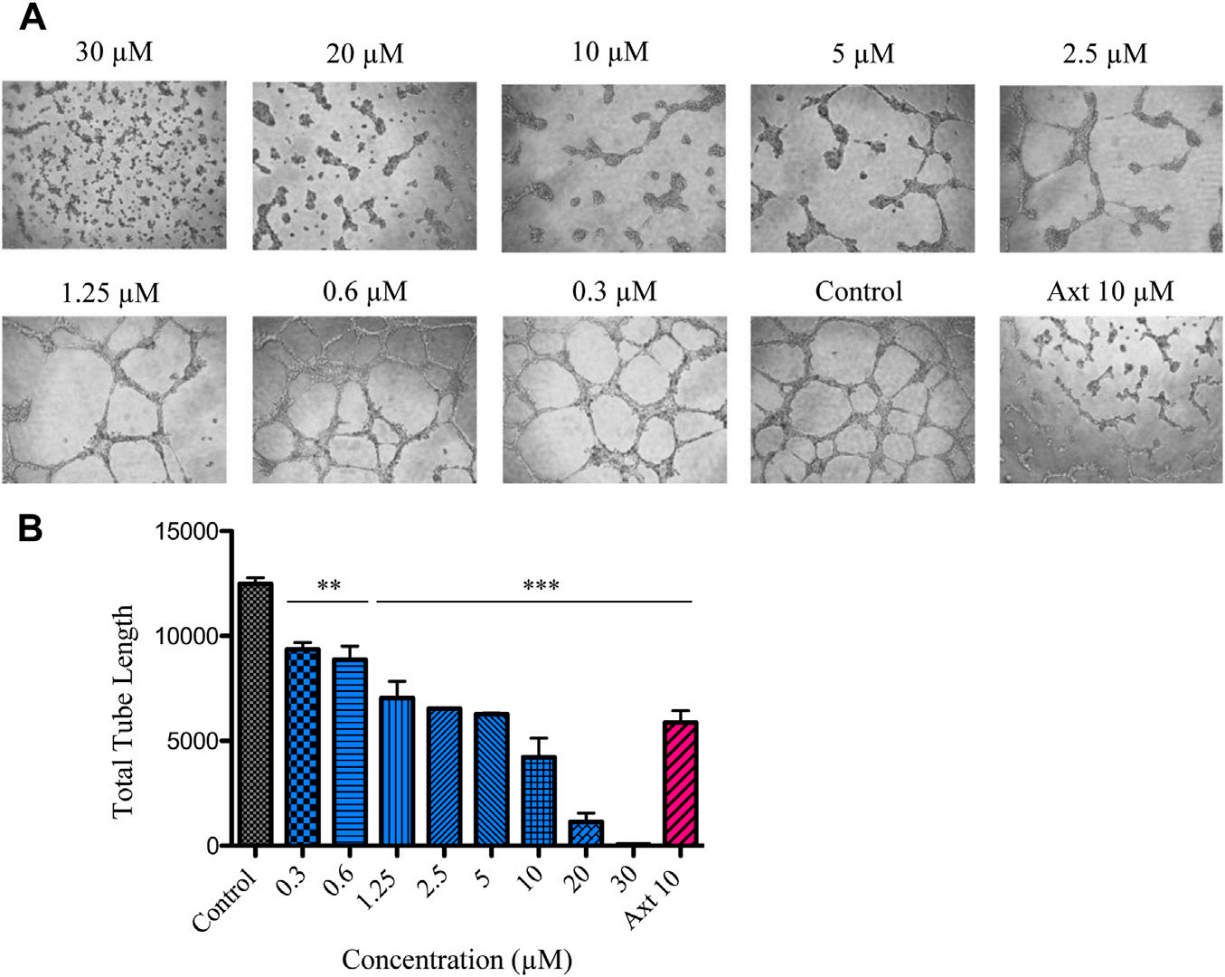
Effect of compound **3** on VEGF-induced tube formation in BAECs. **(A)** Representative images of the tubular networks after treatment with different concentrations of compound **3** (0.3–30 μM) or axitinib (Axt, 10 μM). **(B)** The capillary-like structures were examined and the tube length was quantified using ImageJ software. The bars show the total tube length expressed as the mean ± SEM. Statistical analysis was performed by ANOVA followed by Dunnett’s post-hoc test compared to the negative control (***p < 0.001, **p < 0.01).

Of these, **1** and **2** were devoid of activity, while compound **3** exerted a remarkable antiangiogenic effect by inhibiting tube formation with an IC_50_ value of 2.7 ± 0.4 μM ( ≈ 0.8 μg/ml, more than 44 times lower than that of the original extract). Interestingly, even at 1.2 μM, its antiangiogenic effect was comparable to that of 10 μM of the potent angiogenesis inhibitor axitinib (*p* > 0.05) (Figures 3A,B).

Axitinib was used as positive control at 10 μM, since, under our experimental settings, this was the minimum effective concentration at which this drug significantly inhibited tube formation. It has been well described that higher concentrations of this drug may be required in this type of assays to achieve activity, due to the presence of serum in the system (Hu-Lowe et al., 2008). Similar effective concentrations were previously reported by other authors in assays performed with human umbilical vein endothelial cells (HUVECs) (Sp et al., 2017; Mongiardi et al., 2019).

In agreement with the lack of cytotoxic effect of the *T. minuta* ethanol extract, MTT experiments showed that the active principle tested at 30 μM did not interfere with normal cell proliferation in the time lapse of the experiment.

### Compound **3** inhibited cell invasion without exerting a direct cytotoxic effect

**FIGURE 4:**
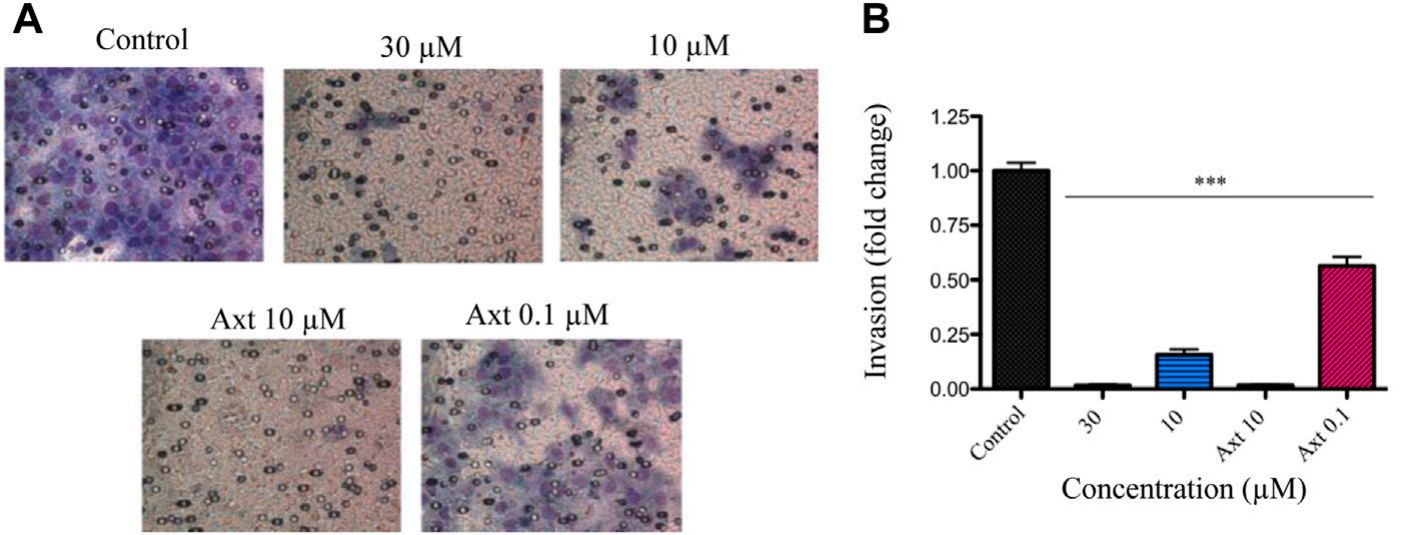
Effect of compound **3** on VEGF-induced invasion in BAECs.BAEC cells were treated with compound **3** (30 and 10 μM) or axitinib (Axt, 10 and 0.1 μM) and the Matrigel invasion assay was performed in a 24 h time frame. **(A)** The images display representative fields of invading cells. **(B)** Graphs show the percentage of invading cells per field relativized to the control. Results are expressed as mean ± SEM. Statistical analysis was performed by ANOVA followed by Dunnett’s post-hoc test compared to negative control (***p < 0.001).

Angiogenesis is a multistep process that involves the dissolution of the basal membrane, increased vascular permeability, and the degradation of the extracellular matrix, allowing endothelial cell migration, invasion, proliferation, and tube formation (Gacche and Meshram, 2014). To gain further insight into the antiangiogenic effect of compound **3**, its influence on endothelial cell invasion was evaluated. Invasiveness of BAEC cells was markedly reduced, with only 1.6 ± 0.4 and 15.7 ± 2.5% of invasive cells when the compound was tested at 30 and 10 μM, respectively (Figures 4A,B).

**FIGURE 5:**
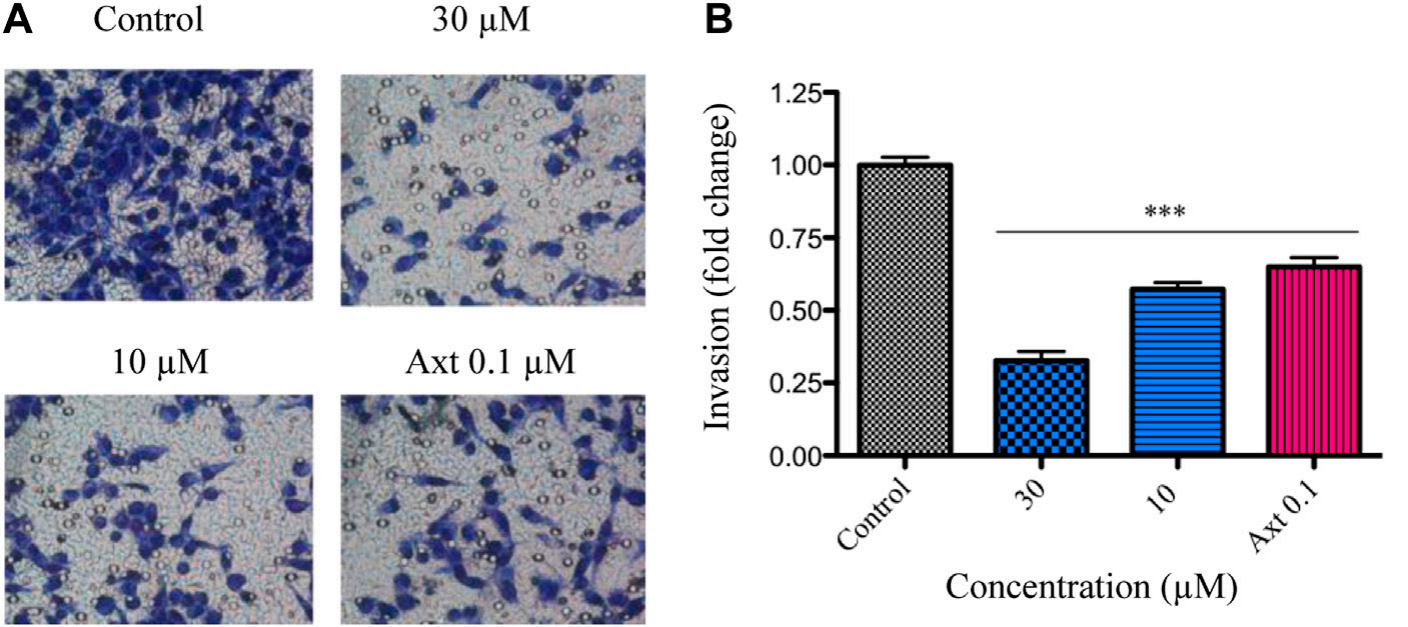
MDA-MB-231 cells were treated with compound **3** (30 or 10 µM) or axitinib (Axt, 0.1 μM), and the Matrigel invasion assay was performed in a 16 h time frame. **(A)** The images display representative fields of invading cells. **(B)** Graphs show the percentage of invading cells per field relativized to the control. Results are expressed as mean ± SEM. Statistical analysis was performed by ANOVA followed by Dunnett’s post-hoc test (***p < 0.001).

In order to validate the results observed in endothelial cells and study the penetrance of α-terthienylmethanol in the regulation of invasiveness, transwell invasion assays were carried out in the highly aggressive, invasive and poorly differentiated cancer cell line, MDA-MB-231. Compound **3** was able to reduce MDA-MB-231 cell invasion when tested at 30 and 10 μM by 32.7 ± 3.1 and 57.2 ± 2.3%, respectively (Figures 5A,B). MTT experiments demonstrated that the compound at 30 μM did not exert a cytotoxic effect against this cell line in the time lapse of the experiment. Taken together, these results were consistent with those observed in the BAEC endothelial cells.

### Compound **3** impairs angiogenesis targeting mainly PKC-α and -β2 isozymes

*In silico* experiments were conducted to study the possible mechanisms of inhibition by compound **3** of key proteins that mediate VEGF signaling pathway. As a first approach, we decided to investigate whether the compound could exert a direct inhibitory effect over VEGFR-2, one of the most frequent targets of clinically used drugs (Roskoski, 2008, 2017).

**FIGURE 6:**
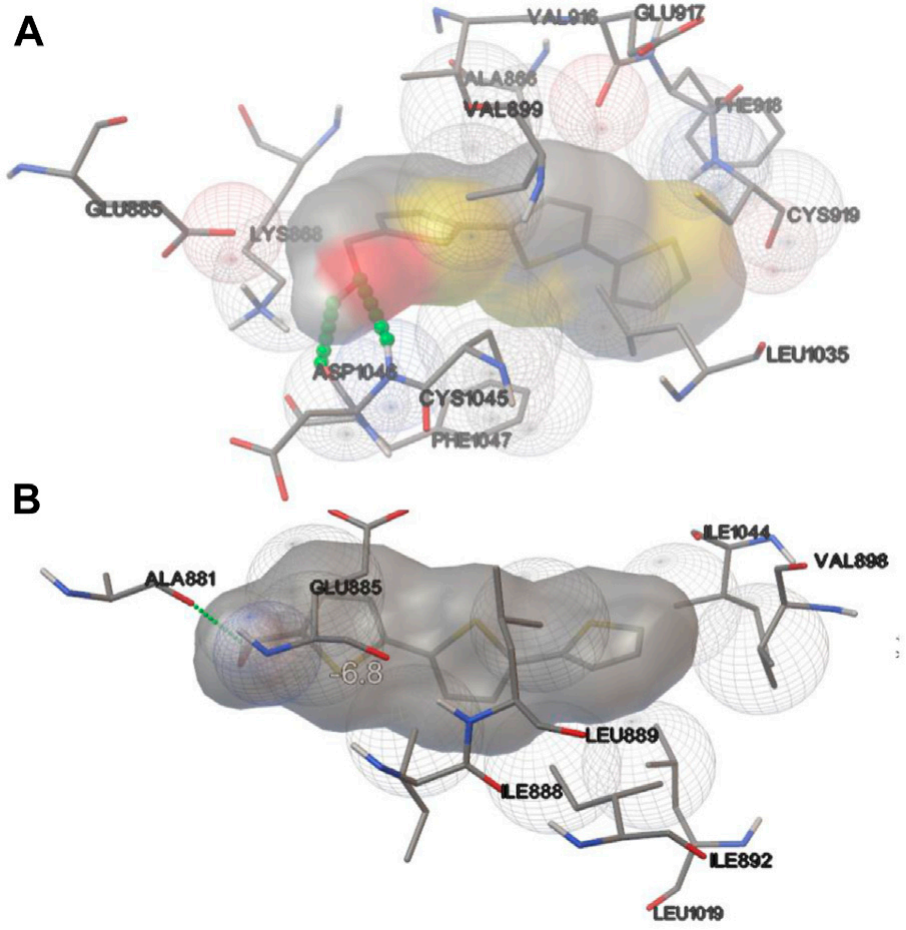
**(A)** Lowest energy docked structure for the VEGFR2/compound **3** complex at the site shared with sorafenib and axitinib. **(B)** A secondary pose at the site HYD-II. Both structures were used as initial geometries for the MD runs.

**FIGURE 7:**
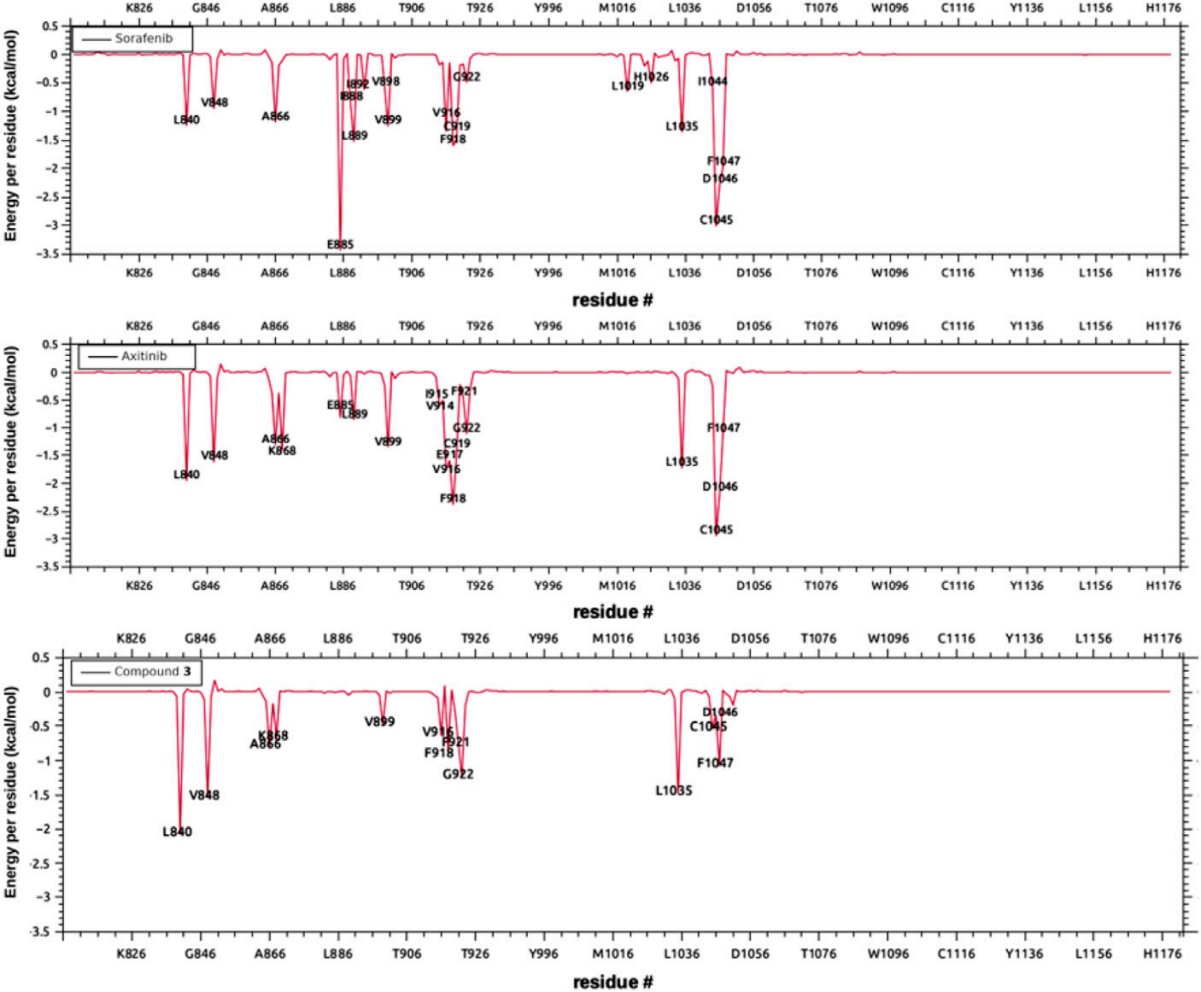
Decomposition on per residue basis of the contribution of each amino acid to the free energy of binding, from top to bottom: sorafenib, axitinib and compound **3**.

**FIGURE 8:**
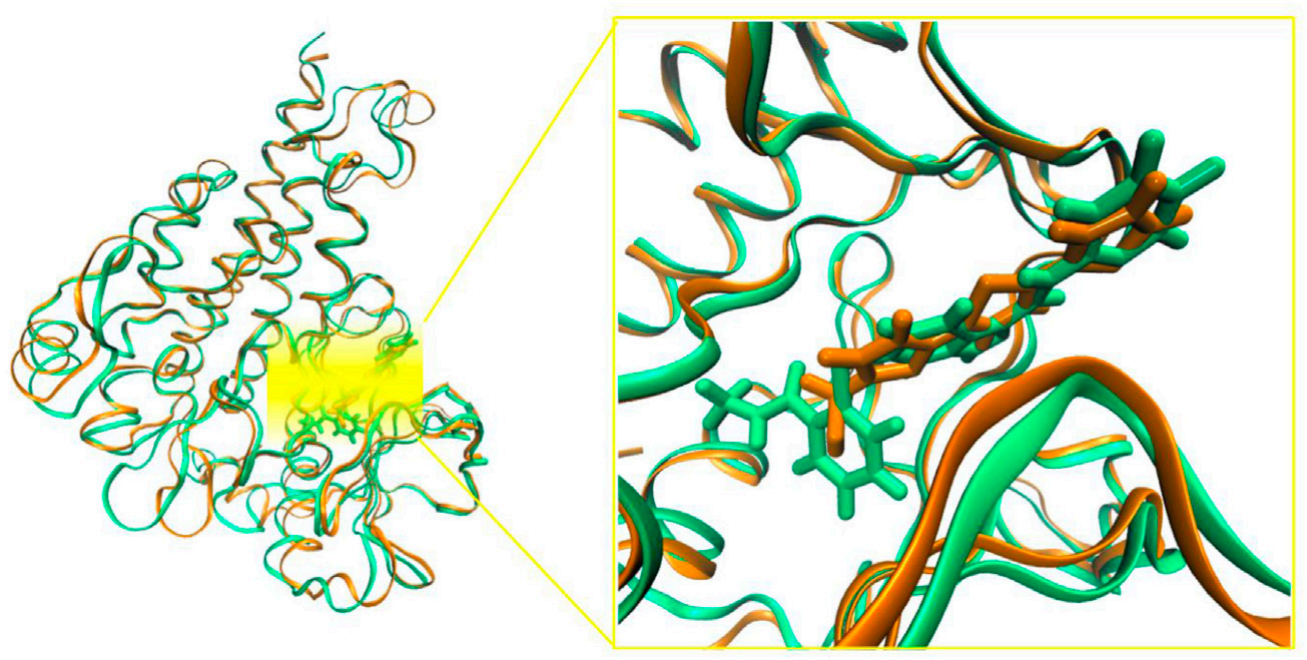
Superimposition of the most populated cluster from the cluster analyses of one representative trajectory of sorafenib (green) and compound **3** (brown). Cluster analyses from the last 60 ns of simulation.

For this purpose, MD simulations were run for the complexes of VEGFR-2 with sorafenib (X-ray reference), axitinib (tested alongside compound **3** and used in this study as a positive control), and the subject compound **3**. The MD simulations of sorafenib were easily equilibrated and the pose in the crystal conserved during 100 ns, thus confirming the reliability of the model (RMSD of the backbone and the sorafenib plus its contacts 5 Å around are shown in Supplementary Figure S2). Compound **3** was simulated both in the lowest energy docked pose, which considerably overlaps with sorafenib, and in the secondary pocket HYD-II, as shown in Figure 6. These trajectories were compared to those obtained for axitinib (experimental reference) and sorafenib (crystallographic reference) in terms of total free energy of binding, Δ*G*_*b*_°, summarized in Table 1, and in terms of the decomposition on a per residue basis (Figure 7). These analyses allow us to compare the relative relevance of each contact along with the time contributing to the overall binding stabilization, and to what extent the dynamic description of the binding modes are similar between the known and the subject inhibitors. The central structure of the most populated cluster obtained from the cluster analysis of one representative trajectory of the VEGFR-2/compound **3** complex is superimposed on the corresponding structure of the cluster for VEGFR-2/axitinib simulation in Figure 8. Both inhibitors clearly overlap in the main site (where the sorafenib was co-crystallized). However, greater insight was obtained from Figure 7, where the decomposition analyses are compared for sorafenib, axitinib and compound **3** complexes in the last part of their equilibrated trajectories. The main residues found involved in the binding of **3** were L840, V868, A866, V899, V916, F918, F921, G922, L1035, C1045 and F1047, most of them hydrophobic in nature, allowing for accommodating the conjugated system of the ligand, and all of them shared with axitinib. However, the charged contacts, E885 and L889, are missing. It was also noted that some other residues had a smaller contribution than in the case of axitinib. Sorafenib revealed a few extra contacts compared to axitinib and even tighter peaks. Similarly, an overall binding energy was found consistently less favored for compound **3** (Table 1), even though it mimics the binding mode of axitinib. These results are not fully consistent with the experimental assays, where axitinib was found at least comparable in terms of antiangiogenic activity. A possible explanation within the hypothesis of inhibition mediated directly by VEGFR-2 may be the interaction of compound **3** in the secondary binding site according to the docking results, which sent it to the HYD-II pocket. This region was also identified as an alternative binding site. The free energy of binding in this hydrophobic site was quite similar (Table 1) to the previous site, i.e., the one which is also preferred by axitinib. In this regard, a two-molecule binding mode, which was also simulated (details in Supplementary Figure S3), could not be discarded. These possibilities may be confirmed or discarded by means of further enzymatic kinetics studies. In the light of the *in silico* studies so far, certain VEFGR-2 inhibitory activity can be ascribed to compound **3**, but there is no clear evidence pointing to it as a powerful VEFGR-2 inhibitor, despite the experimental activity shown in the tube formation assay.

**TABLE 1:** Summary of the free energies of binding (MM-PBSA) from the MD simulations of the VEGFR-2/inhibitor complexes.

Inhibitor	Δ*G*_b_°/(kcal/mol)
Sorafenib	−49.6
Sorafenib (site HYD-II)	−37.9
Axitinib	−41.7
Compound **3**	−21.6
Compound **3** (site HYD-II)	−21.8

In view of these findings, the effect of compound **3** on downstream mediators was considered. Since PKC isozymes α and β2 are targets of this compound (Kim et al., 1998) and considering the crucial role of these kinases as key components of the VEGF driven response through the VEGF-VEGFR2-PLCγ-PKC-ERK1/2 pathway, it was decided to further study the interactions of this molecule with both PKC isozymes.

**FIGURE 9:**
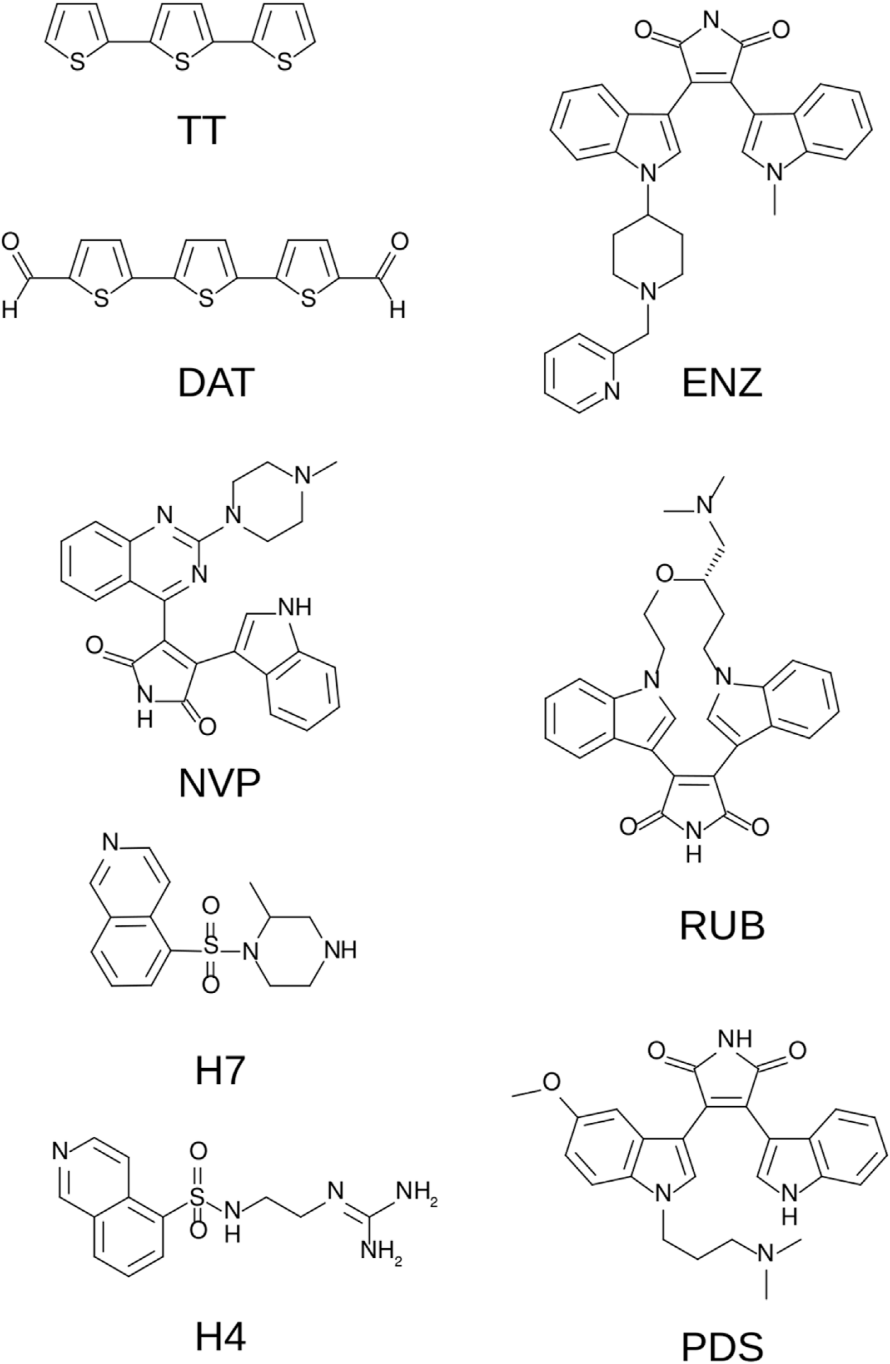
Comparison set for the different PKC (α or β2 isozymes)/inhibitor complexes. Includes terthiophene (TT), its dialdehyde derivative (DAT), enzastaurin (ENZ), NVP, ruboxistaurin (RUB), H7, H4 and PDS (see text).

The PKC-α/compound **3** and PKC-β2/compound **3** complexes were subjected to MD simulations within a thorough comparative study against mild (10 μM) to very powerful (0.1 nM) known inhibitors. With this aim, the comparative set of compounds shown in Figure 9 was built. It includes the crystallographic inhibitors NVP and PDS which were co-crystallized with PKC-α and -β2 isozymes, respectively.

**FIGURE 10:**
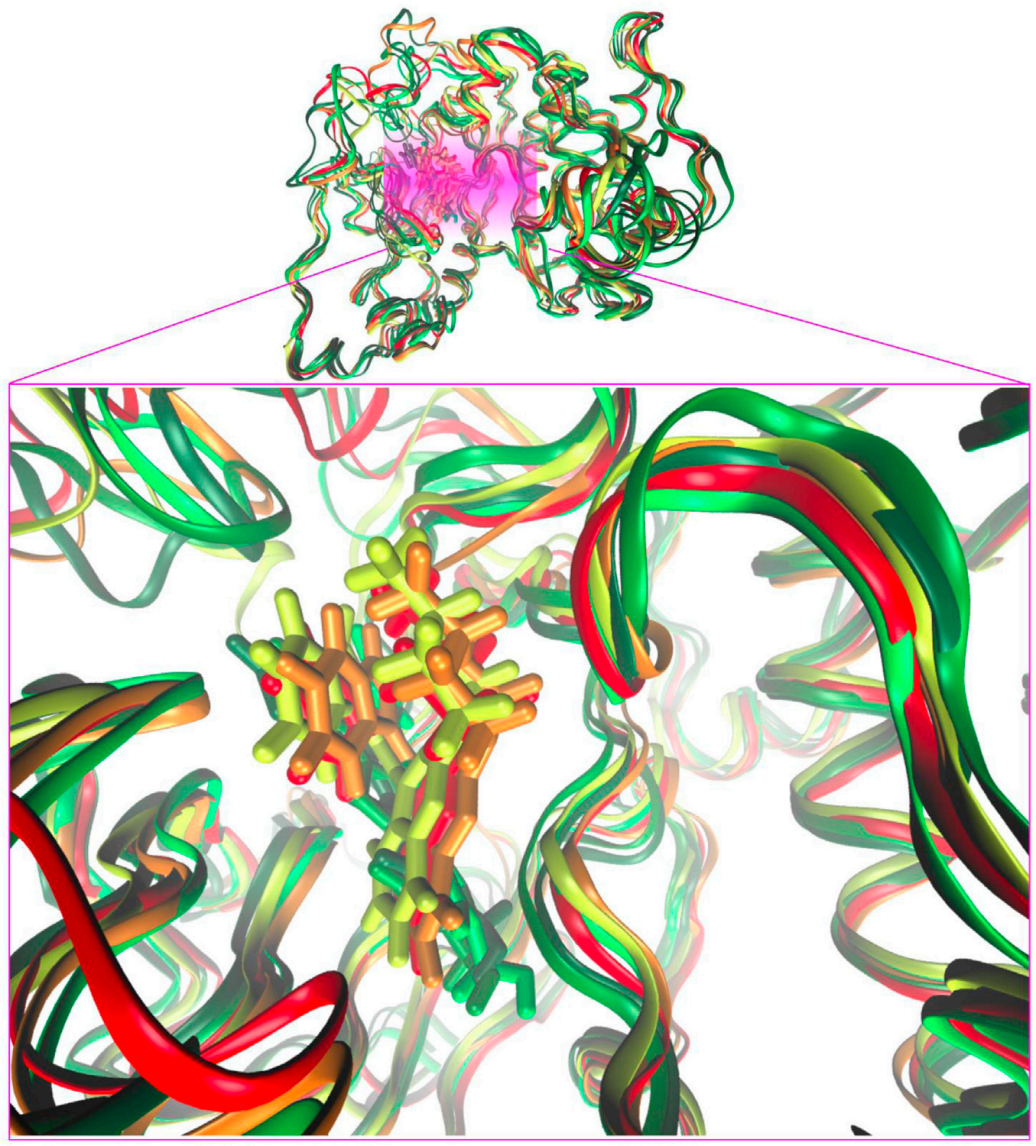
Superimposition of representative snapshots of the most populated clusters of the PKC-α/NVP trajectory (red-orange tones) and the corresponding ones that form the PKC-α/compound **3** (lime-green tones). Cluster analyses from the last 20–100 ns.

**FIGURE 11:**
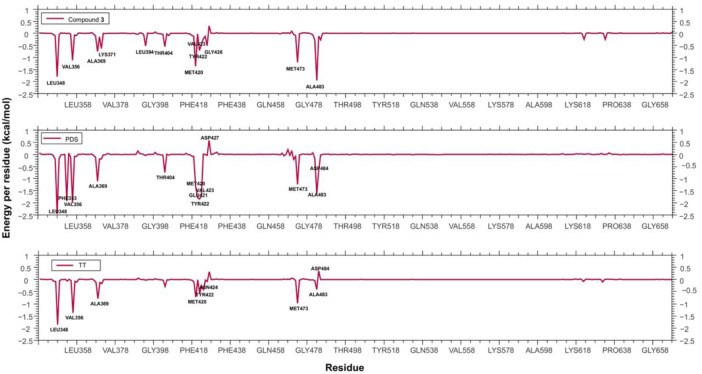
Per residue contributions to the free energy of binding: same as Figure 9 for the case of the complexes PKC-β2/compound **3,** PKC-β2/PDS and PKC-β2/TT.

It was first demonstrated that our subject compound clearly mimics both the binding site and the dynamic behavior of the crystallographic inhibitors NVP for PKC-α, and PDS for PKC-β. Figure 10 shows a superimposition of the three most populated clusters of compound **3** and the three corresponding to the crystallographic inhibitor NVP (similar comparison for the β2/PDS and β2/compound 3 complexes available as Supplementary Figure S4). The contributions to the binding energy decomposed by residues are compared in Figure 11 for the case of PKC-β2 and one of its most powerful inhibitors (PDS), our subject compound and one of the less active of the set (TT, 10 μM) (a similar comparison for the α isozyme involving NVP (0.95 nM) and compound **3** is shown in Supplementary Figure S5). The Δ*G*°_*b*_’s obtained for each PKC/inhibitor complex are summarized in Table 2. The cavity occupied by **3** in the latter involved the hydrophobic residues L348, V356, A369, L394, T404, M420, Y422, G425, M473, and A483, and the charged L371. All of them shared with PDS except L371, the main difference being the lack of the strong contact with E421 observed for PDS.

**TABLE 2:** Summary of the Δ*G*_b_° from the MD simulations and experimental IC_50_ of each inhibitor against each isozyme.

Inh/PKC	ΔG°/(kcal/mol)	IC_50_/μM	Log(IC_50_/M)	References
NVP/α	−38.4	0.00095	−9.0223	Evenou et al. (2009)
PDS/β	−41.2	0.002	−8.6990	Young et al. (2005)
RUB/β	−38.8	0.0059	−8.2291	Tang et al. (2008)
ENZ/β	−41.3	0.006	−8.2218	Graff et al. (2005)
PDS/α	−38.1	0.007	−8.1549	Young et al. (2005)
DAT/αβ	−25.2	0.3	−6.5229	Kim et al. (1998)
RUB/α	−32.6	0.36	−6.4437	Tang et al. (2008)
Compound **3**/α	−26.7	4.0	−5.3979	Kim et al. (1998)
Compound **3**/β	−32.3			
H4/αβ	−22.8	10	−5.0000	Kinsella et al. (1992)
TT/αβ	−16.7	10	−5.0000	Kim et al. (1998)
H7/αβ	−19.9	40	−4.3979	Kinsella et al. (1992)

α and β: denotes that the experiments were 1:1 mixture; in those cases, the Δ*G*_b_° reported was taken as an average of the runs with the α and β isozymes.

**FIGURE 12:**
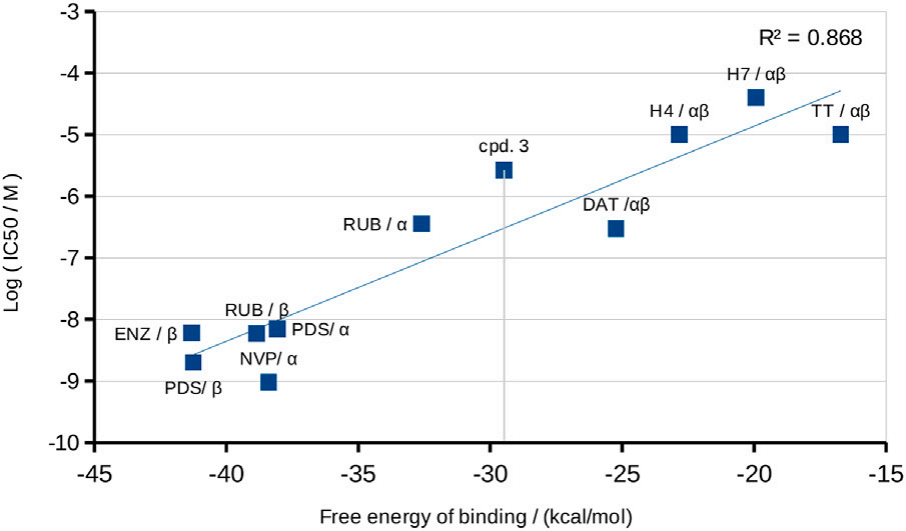
Correlation between the Δ*G*_b_° from the MD simulations and the experimental IC_50_ for the comparison set of molecules (references in Table 2).

According to these comparisons, compound **3** would be placed between the mild and the most powerful PKC inhibitors. A robust argument reinforcing the reliability of these MD estimations to the Δ*G*°_*b*_’s is illustrated in Figure 12, where the logarithmic correlation between the free energies of binding and the experimental IC_50_ obtained from different experimental sources is shown.

### Compound **3** had no toxic effect on peripheral blood mononuclear cells and did not affect erythrocyte membrane integrity

Even at the maximum concentration of 100 μM the compound resulted innocuous to both PBMCs and erythrocytes. These results suggest that the compound is devoid of toxicity.

### Compound **3** showed promising pharmacological properties and drug-likeness parameters

The determination of the pharmacological properties and evaluation of drug-likeness parameters of compound **3** was performed using the online tool SwissADME (Daina et al., 2017). Interestingly, the thiophene showed high gastrointestinal absorption, which is very encouraging in terms of oral administration. In line with this, the tested compound showed a good bioavailability score (55%).

The compound followed the Lipinski rule of five, Ghose, Veber, Egan and Muegge, with no violation of any of these filters. Lipinski rules of five predict that poor absorption or permeation is more likely to occur when there are more than 5 H-bond donors, 10 H-bond acceptors, the molecular weight is greater than 500 and the calculated Log P (CLogP) is greater than 5 or the Moriguchi Log P (MLogP) is greater than 4.15 (Lipinski et al., 2001). In association with this, compound **3** has a molecular weight of 278.41 g/mol; presents one H-bond acceptor (oxygen at OH group) and one H-bond donor (H at the OH group). The consensus CLogP (average of all predictions) is 4.35 with a MLogP of 2.57.

The compound did not inhibit the cytochrome P450 subunits CYP2D6 and CYP3A4 (vital enzymes involved in drug metabolism), which confirms phase I metabolism (Singh et al., 2021). Additionally, the terthiophene was unable to permeate through the blood-brain barrier. It is important to highlight that the thiophene did not present any potentially problematic substructure (i.e., fragments potentially toxic, chemically reactive, metabolically unstable, or related to a poor pharmacokinetics) which was reflected in the absence of PAINS (Baell and Holloway, 2010) or Brenk alerts (Brenk et al., 2008).

## Discussion

Pathological angiogenesis arises as a common denominator of different life-threatening conditions with a strong implication in carcinogenesis, cancer progression and metastasis (Aguilar-Cazares et al., 2019; Quintero-Fabián et al., 2019). These statements are important in the field of drug discovery for the development of blockbuster medications (Folkman, 2007).

After evaluating the antiangiogenic activity of 40 plant-derived ethanol extracts, those from *A. citriodora, A. gratissima, B. pilosa, H. heptaphyllus* and *T. minuta* resulted the most effective for inhibiting endothelial cell tube formation. Zihlif et al. (2012) reported a selective antiangiogenic effect for *A. citriodora* extract at 100 μg/ml using the aortic ring assay with no cytotoxic activity against HUVEC, PFL and MCF7 cells at the mentioned concentration. As far as we know, there are no previous reports about the antiangiogenic effect of *A. gratissima*. Some of the most studied components of *H. heptaphyllus*, dehydro-α-lapachone and β-lapachone, have also demonstrated antiangiogenic activity (Kung et al., 2007; Garkavtsev et al., 2011). The highest inhibitory values in the screening were those obtained for *B. pilosa* and *T. minuta* (100% and 95.5 ± 4.5%, respectively). The antiangiogenic effect of *B. pilosa* was attributed to a novel polyacetylene compound known as 1,2-dihydroxy-5(*E*)-tridecene-7,9,11-triyne (Wu et al., 2007). There are still no precedents regarding the antiangiogenic activity of *T. minuta*, and therefore, together with the potent effect demonstrated by its extract, this species was selected to be submitted to bioassay-guided fractionation. From this process, the terthiophene **3** was isolated as effective for inhibiting angiogenesis. The presence of this compound has been previously described in the *Eclipta* species, such as *E. alba*, *E. erecta* and *E. prostrata* (Kagan, 1991)*.*

This work represents the first report of the antiangiogenic activity of compound **3**. The compound not only efficiently inhibited tube formation, but also transwell cell invasion, without exerting toxic effect on the assayed cell lines (BAEC and MDA-MB-231). Interestingly, there are previous reports of its antitumoral effect in human ovarian and endometrial cell lines, with IC_50_ values ranging from 0.35 to 18.82 μM (Lee et al., 2015; Preya et al., 2017). This background indicates the potential of this compound to simultaneously affect key hallmarks of tumor progression, such as angiogenesis and neoplastic cell development and invasiveness (Hanahan and Weinberg, 2011).

Regarding the molecular mechanism of action, *in silico* experiments indicated that the compound was able to bind to VEGFR-2, sharing some common features with axitinib and sorafenib, but with a lower affinity. Therefore, we decided to explore other possible targets located downstream of this master receptor. It is widely established that the activation of VEGFR-2 in response to VEGF activates crucial cellular pathways. Among these, the PLCγ-PKC-ERK1/2 pathway is involved in cell proliferation, migration and homeostasis (Simons et al., 2016). Interestingly, Kim et al. (1998) reported compound **3** as part of a novel series of PKC inhibitors. In this study, the activity was measured *in vitro* using a 1:1 mixture of recombinant PKC-α and -β2 isozymes. The terthiophene was able to inhibit both kinases with an IC_50_ of 4 μM (Kim et al., 1998). In addition, Xia et al. (1996) showed that VEGF induces concentration- and time-dependent activation of PKC isozymes α and β2 in BAEC cells. It is also important to remark that the activation of PKCs is a key determining step in endothelial tube formation in the basement membrane matrix (Kinsella et al., 1992). Under this scenario and in agreement with the experimental model used in the present work, the inhibitory activity of compound **3** on the PKC isozymes α and β2, and the consequent impairment of the VEGF-VEGFR-2-PLC**γ**-PKC-ERK1/2 axis emerged as the main molecular mechanism that could explain the antiangiogenic effect of the thiophene described here. In agreement with the proposed mechanism, compound **3** strongly inhibited the transwell invasiveness of BAEC cells and of the highly metastatic cell line, MDA-MB-231. Indeed, Llorens et al. (2019) recently unveiled the molecular mechanisms by which PKC-α modulates epithelial-to-mesenchymal transition (EMT) and the invasiveness of breast cancer cells through regulation of ZEB1 expression. In this context, together with the impairment of tube formation, the inhibition of cell invasion on both cell lines could be explained mainly in terms of PKC inhibition.

An important mass of 100 ns MD simulations with different known inhibitors of PKC-α and -β2 was used as a quantitative reference framework to place the power of compound **3** as a suitable inhibitor for these targets. The difference between the Δ*G*_b_° obtained for α and β2 isozymes (Table 2) evidenced some preference for the β2 isoform. Even though strong evidence points to PKC as the main target of compound **3**, a certain inhibitory activity against VEGFR-2 cannot be discarded. In this case, it would be less important than that for the PKC’s. Interestingly, a combination of both effects would make the compound still more promising.

## Conclusion

The present work is the first study to describe the antiangiogenic activity of *T. minuta* and its active principle, compound **3**. The molecular interactions between this thiophene and key players of the angiogenic response, such as VEGFR-2 receptor and PKC-α and -β2 isozymes, were exhaustively investigated by means of docking and MD simulations. The terthiophene emerged as a suitable inhibitor for PKC-α and -β2, with a preference of about 6 kcal/mol for the PKC-β2 isozyme. The inhibitory activity on the latter was proved to be in the middle between those mild and very strong inhibitors. Nevertheless, the remarkable antiangiogenic activity observed in the functional assays suggests that a weak inhibitory activity against VEGFR-2 should not be underestimated since a combined mechanism could be implicated, making this compound still more promising.

As far as we know, there has been no previous description of an antiangiogenic effect exerted by a terthiophene scaffold, which makes its further investigation even more interesting. Despite more studies being needed, these results position this natural compound as a potential candidate for the development of new angiogenesis inhibitors.

## Data Availability

The original contributions presented in the study are included in the article/Supplementary Materials, further inquiries can be directed to the corresponding authors.

## Abbreviations

AXT, Axitinib; BAEC, Bovine Aortic Endothelial Cells; DAT, Dialdehyde Derivative; ENZ, Enzastaurin; ERK, Extracellular Signal-regulated Kinase; GB, Generalized Born; H4, N-(2-guanidinoethyl)isoquinoline-5-sulfonamide; H7, 1-(5-Isoquinolinesulfonyl)-2-methylpiperazine; HYD, Hydrophobic Pocket; IC_50_, Half-maximal Inhibitory Concentration; MD, Molecular Dynamics; MDA-MB-231, MD Anderson-Metastatic Breast-231 Cells; NTV, (constant) Number of Particles Temperature and Volume; NTP, (constant) Number of Particles Temperature and Pressure; NVP, NVP-AEB071: 3-(1H-indol-3-yl)-4-[2-(4-methylpiperazin-1-yl)quinazolin-4-yl]-1H-pyrrole-2,5-dione; PB, Poisson-Boltzman; PBMC, Peripheral Blood Mononuclear Cells; PDB, Protein Data Bank; PDS, Sotrastaurin (3-(1H-Indol-3-yl)-4-[2-(4-methyl-1-piperazinyl)-4-quinazolinyl]-1H-pyrrole-2,5-dione); PKC, Protein Kinase C; PLC, Phospholipase C; PlGF, Placental Growth Factor; RMSD, Root Mean Square Deviation; RUB, Ruboxistaurin; SUR, Suramin sodium; TT, Terthiophene; VEGF, Vascular Endothelial Growth Factor; VEGFR, Vascular Endothelial Growth Factor Receptor.
