# Supplementary material for: The thiophene α-terthienylmethanol isolated from Tagetes minuta inhibits angiogenesis by targeting protein kinase C isozymes α and β2
Source: Front Pharmacol. 2022 Oct 12;13:1007790. doi: 10.3389/fphar.2022.1007790 (PMC9597362; doi:10.3389/fphar.2022.1007790)
Supplement: Supplementary file 1 [file DataSheet1.docx]

1. **Table S1.**
2. Plants from central Argentina screened for antiangiogenic activity

| Plant species | Family | Common name | Yield (%) | Status | Voucher:  UCCOR number | % Tube Inhibition |
| --- | --- | --- | --- | --- | --- | --- |
| *Adesmia muricata* (Jacq.) DC. var*. dentata* (Lag.) Benth | Fabaceae | - | 6.3 | N | 438 | 10.25 ± 4.90 |
| *Aloysia citrodora*  Palau | Verbenaceae | cedrón | 2.4 | N | 262 | 75.50 ± 5.50 |
| *Aloysia gratissima* (Gillies & Hook. ex Hook.) Tronc. | Verbenaceae | palo amarillo | 1.7 | N | 3 | 80.33 ± 4.67 |
| *Amelichloa brachychaeta* (Godr.) Arriaga & Barkworth | Poaceae | - | 5.5 | N | 454 | 7.75 ± 2.29 |
| *Amphilophium carolinae* (Lindl.) L. G. Lohmann | Bignoniaceae | peine de mono | 7.4 | N | 121 | 6.00 ± 1.00 |
| *Anemia tomentosa* (Savigny) Sw*.* | Schizaceae | doradilla | 1.9 | N | 228 | 0 |
| *Araujia brachystephana* (Griseb.) Fontella & Goyder | Apocynaceae | doca, tasi | 5.5 | N | 155 | 20.00 ± 2.00 |
| *Atamisquea emarginata* Miers ex Hook. & Arn. | Capparaceae | atamisqui, matagusanos | 3.15 | N | 188 | 19.00 ± 3.00 |
| *Bidens pilosa* L. | Asteraceae | amor seco | 4.8 | N | 213 | 100 ± 0.00 |
| *Buddleja cordobensis* Griseb | Buddlejaceae | - | 6.3 | E | 196 | 0 |
| *Condalia microphylla* Cav. | Rhamnaceae | piquillín | 1.6 | N | 180 | 20.00 ± 1.00 |
| *Cortaderia speciosa* (Nees & Meyen) Stapf | Poaceae | cortadera | 3.5 | N | 318 | 0 |
| *Croton lachnostachyus* Baill. | Euphorbiaceae | botonillo | 3.5 | N | 187 | 11.50 ± 1.71 |
| *Cynoglossum amabile* Stapf & J.R. Drumm. | Boraginaceae | - | 2.7 | Adv. | 207 | 35.00 ± 1.00 |
| *Handroanthus heptaphyllus* (Vell.) Mattos | Bignoniaceae | lapacho rosado | 2.6 | N | 502 | 82.00 ± 2.00 |
| *Lessingianthus mollissimus* (Hook. & Arn.) H. Rob. | Asteraceae | - | 2.0 | E | 204 | 40.50 ± 8.50 |
| *Ligaria cuneifolia* (Ruiz & Pav.) Tiegh. | Loranthaceae | liga roja | 4.4 | N | 219 | 0 |
| *Ligustrum lucidum* W. T. Aiton | Oleaceae | siempreverde | 9.0 | Adv. | 532 | 0 |
| *Lippia turbinata* Griseb. | Verbenaceae | poleo | 4.8 | N | 206 | 15.00 ± 8.00 |
| Marrubium vulgare L. | Lamiaceae | malvarrubia | 3.2 | Adv. | 223 | 6.50 ± 0.50 |
| *Melissa officinalis* L. | Lamiaceae | melisa | 1 | Adv. | 240 | 27.50 ± 2.50 |
| Minthostachys verticillata (Griseb.) Epling | Lamiaceae | peperina | 3.6 | E | 125 | 26.50 ± 2.50 |
| Monnina dictyocarpa Griseb. | Polygalaceae | quelén | 6.4 | E | 277 | 8.50 ± 5.50 |
| *Pascalia glauca* Ortega | Fabaceae | sunchillo, asolador | 13.6 | E | 185 | 0 |
| *Plantago australis* Lam. ssp. hirtella (Kunth) Rahn | Plantaginaceae | - | 6.2 | N | 494 | 0 |
| *Podranea ricasoliana* (Tanfani) Sprague | Bignoniaceae | trompeta rosada | 4.5 | Adv. | 164 | 46.00 ± 2.00 |
| *Polystichum montevidense* (Spreng.) Rosenst. | Dryopteridaceae | - | 3.6 | N | 450 | 0 |
| *Porlieria microphylla* (Baill.) Descole, O´Donell & Lourteig | Zygophyllaceae | cucharero | 1.1 | N | 154 | 18.00 ± 5.00 |
| *Rhynchosia diversifolia* Micheli | Fabaceae | - | 4.1 | N | 394 | 15.67 ± 7.31 |
| *Rupechtia apetala* Wedd. | Polygonaceae | manzano del campo | 1.9 | N | 151 | 10.00 ± 2.00 |
| Schizachyrium condensatum (Kunth) Nees | Poaceae | paja colorada | 1.9 | N | 225 | 7.67 ± 6.96 |
| *Senecio madagascariensis* Poir. | Asteraceae | - | 4.9 | Adv. | 208 | 0 |
| *Senecio vira-vira* Hieron | Asteraceae | viravira | 3.9 | N | 181 | 4.33 ± 1.86 |
| *Solanum argentinum* Bitter & Lillo | Solanaceae | duraznillo blanco | 5.4 | N | 34 | 0 |
| *Solanum sisymbriifolium* Lam. | Solanaceae | espina colorada | 2.5 | N | 199 | 9.00 ± 6.00 |
| *Tagetes minuta* L. | Asteraceae | suico | 2.6 | N | 138 | 95.5 ± 4.5 |
| *Thelesperma megapotamicum* (Spreng.) Kuntze | Asteraceae | arnica | 6.1 | N | 303 | 12.00 ± 4.00 |
| *Tradescantia fluminensis* Vell. | Commelinaceae | - | 4.3 | N | 443 | 14.33 ± 0.67 |
| Vachellia aroma (Gillies ex Hook. & Arn.) Seigler & Ebinger | Fabaceae | tusca | 29.6 | N | 242 | 0 |
| *Verbesina encelioides* (Cav.) Benth. & Hook. f. ex A. Gray | Asteraceae | - | 4.9 | N | 364 | 14.00 ± 3.00 |

1. Adv: adventive; E: endemic; N: native


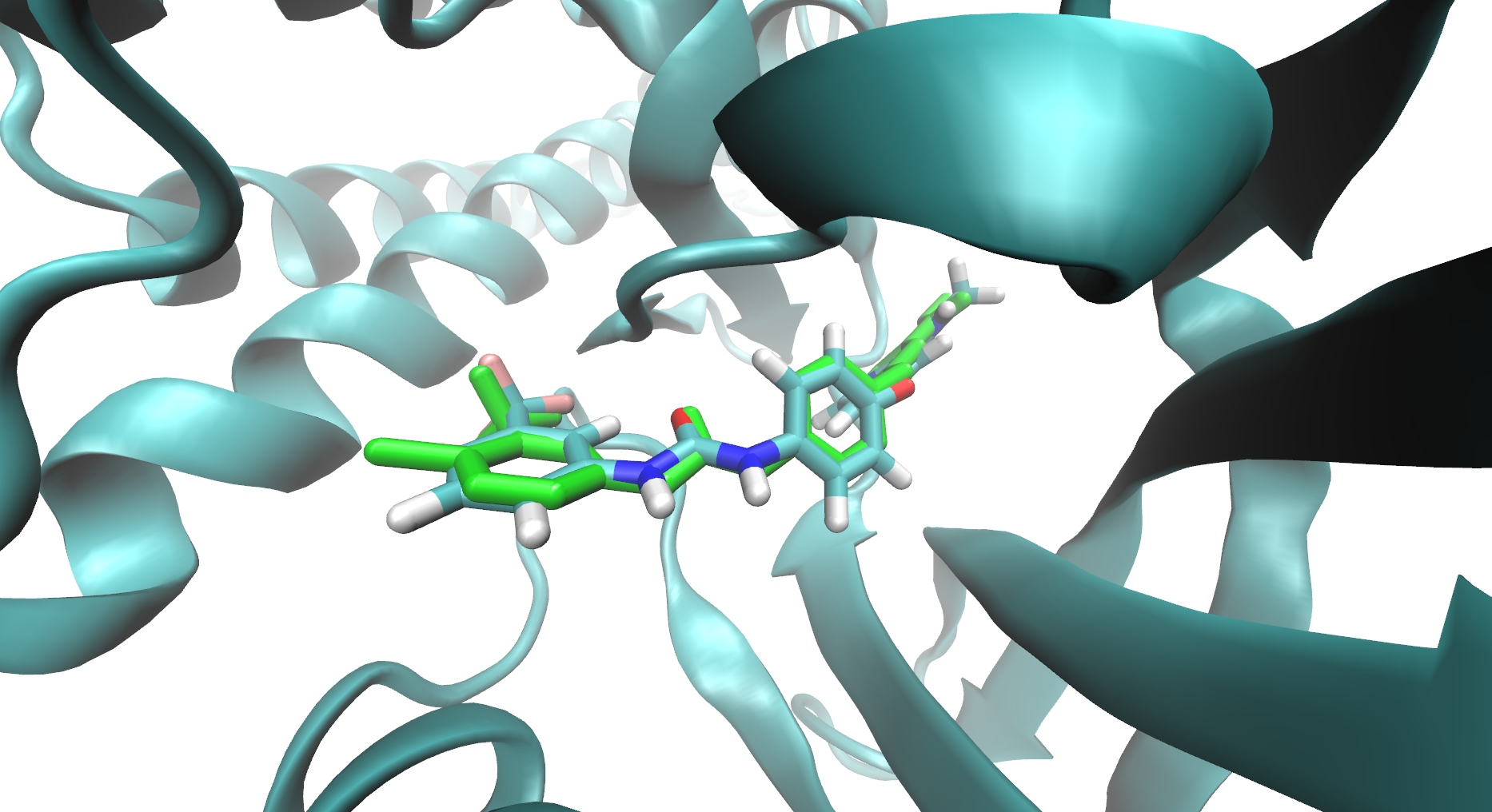


**Figure S1** Superimposition of re-docked (colors according to the atom types) and crystallized (green) poses of sorafenib into the VEGFR-2. Blind docking was used as check for the docking protocol for all co-crystallized structures used.

**
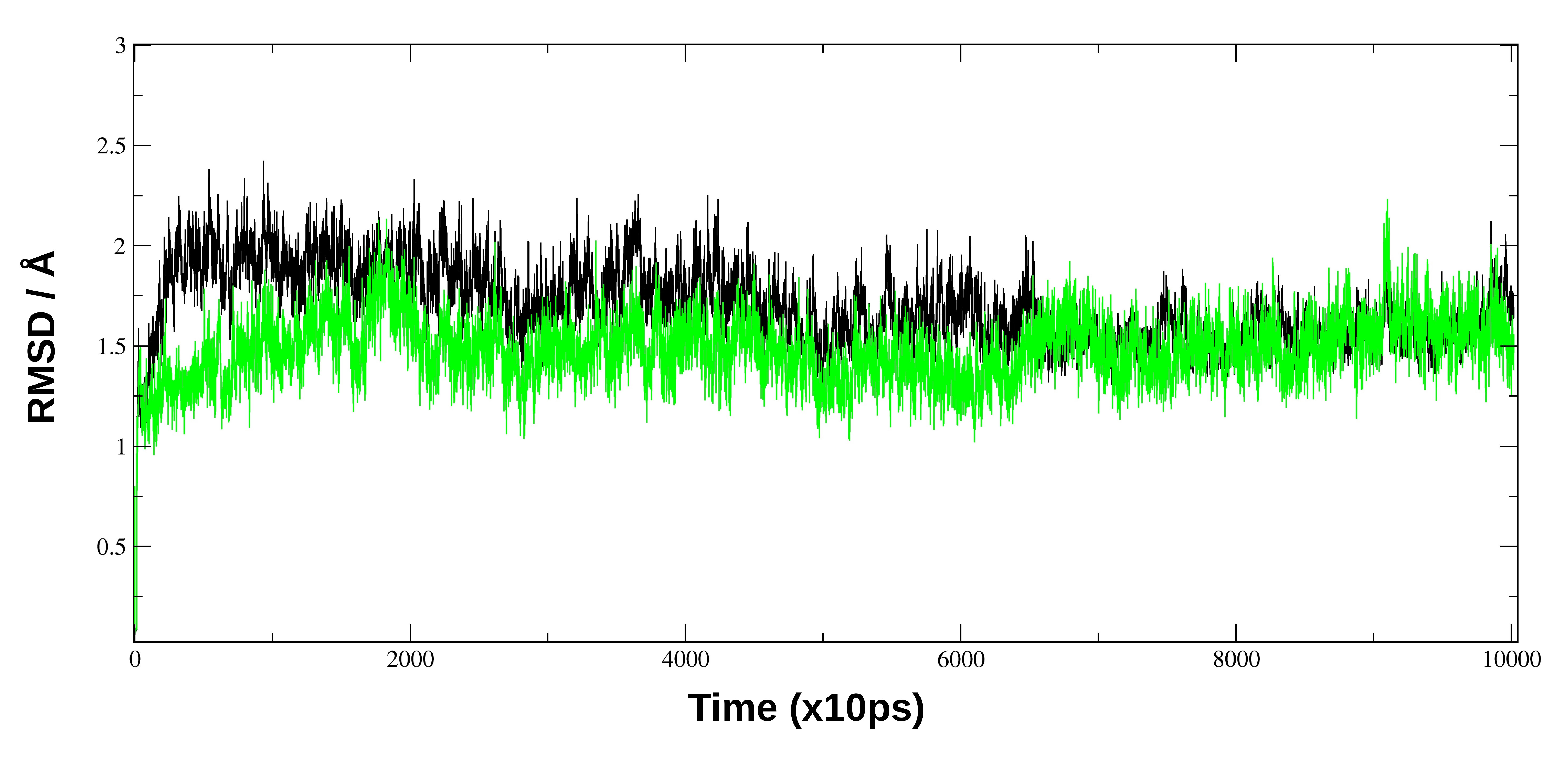
Figure S2** RMSD of VEGFR-2 (backbone, black) and sorafenib plus its contacts 5 Å around (green) during 100 ns of simulations.

**
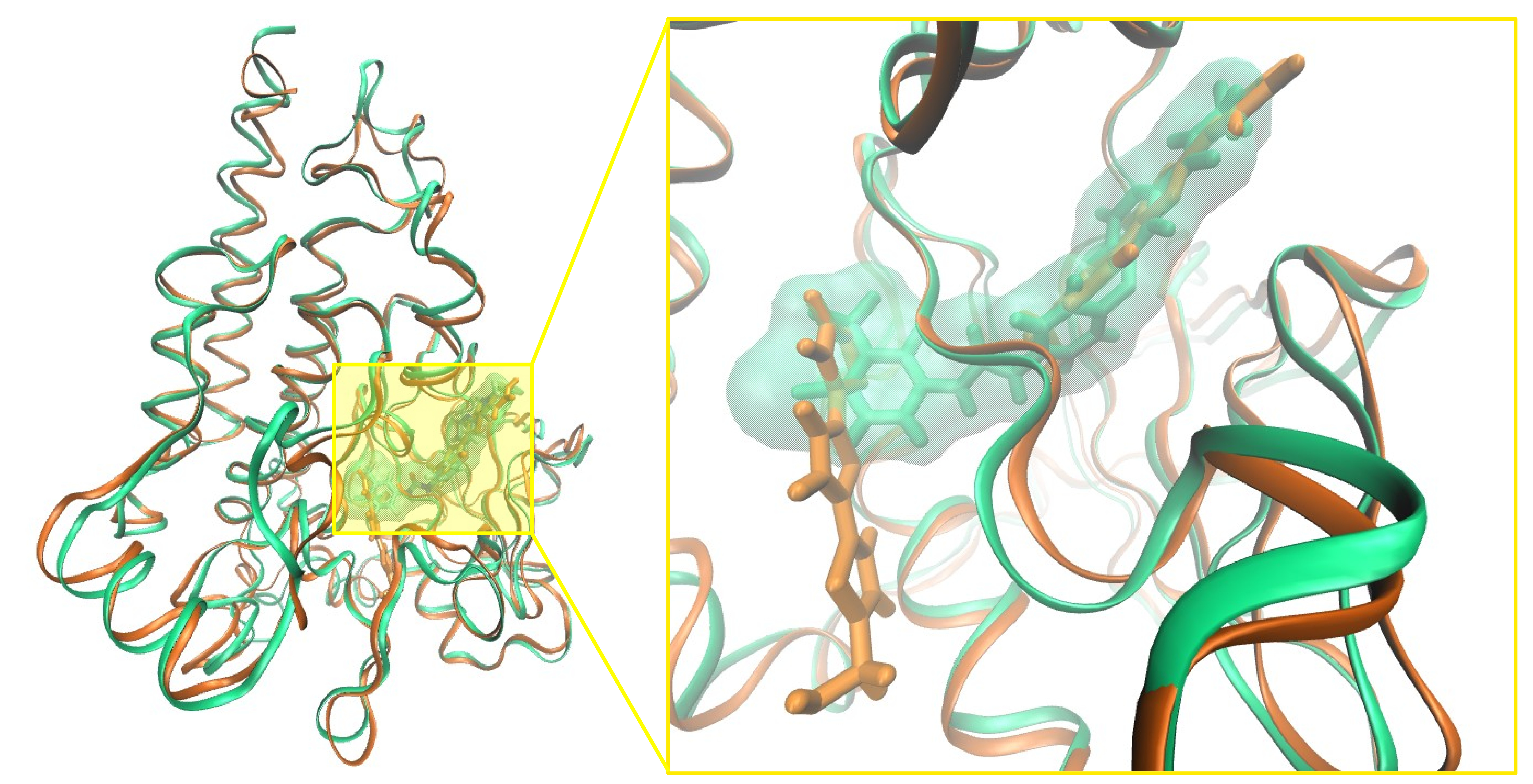
Figure S3** Superimposition of the most populated cluster of one representative trajectory of sorafenib (green) and the most populated cluster of one representative trajectory of two molecules-binding mode of compound **3** (brown). Cluster analyses from the last 50 ns of simulation.


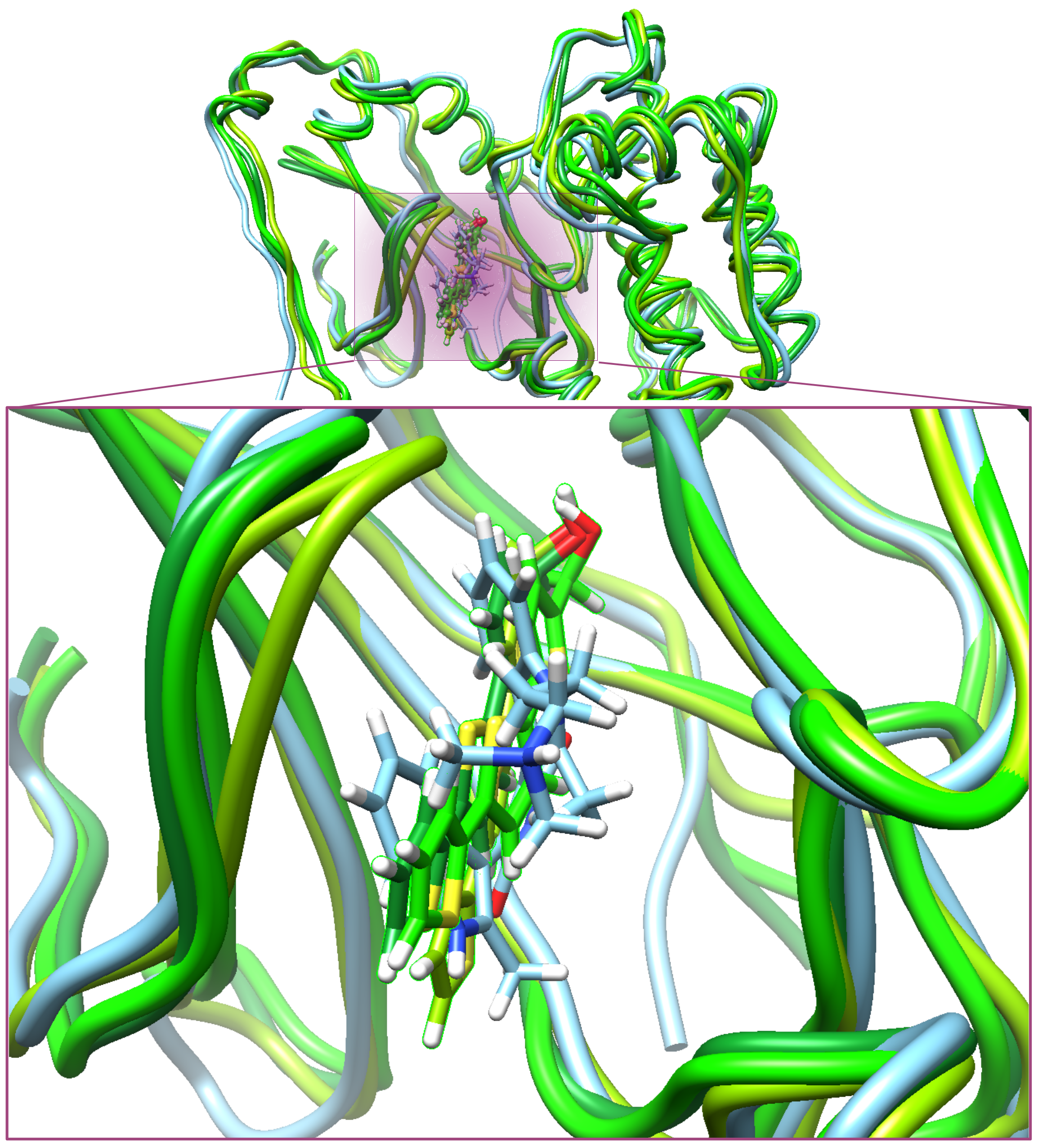


**Figure S4** Superimposition of the most populated cluster of the PKC β / PDS complex (cyan) and the three most populated clusters of the PKC β / compound **3** (green tones) after cluster analyses of the MD runs at the last 50 ns of simulation.

**
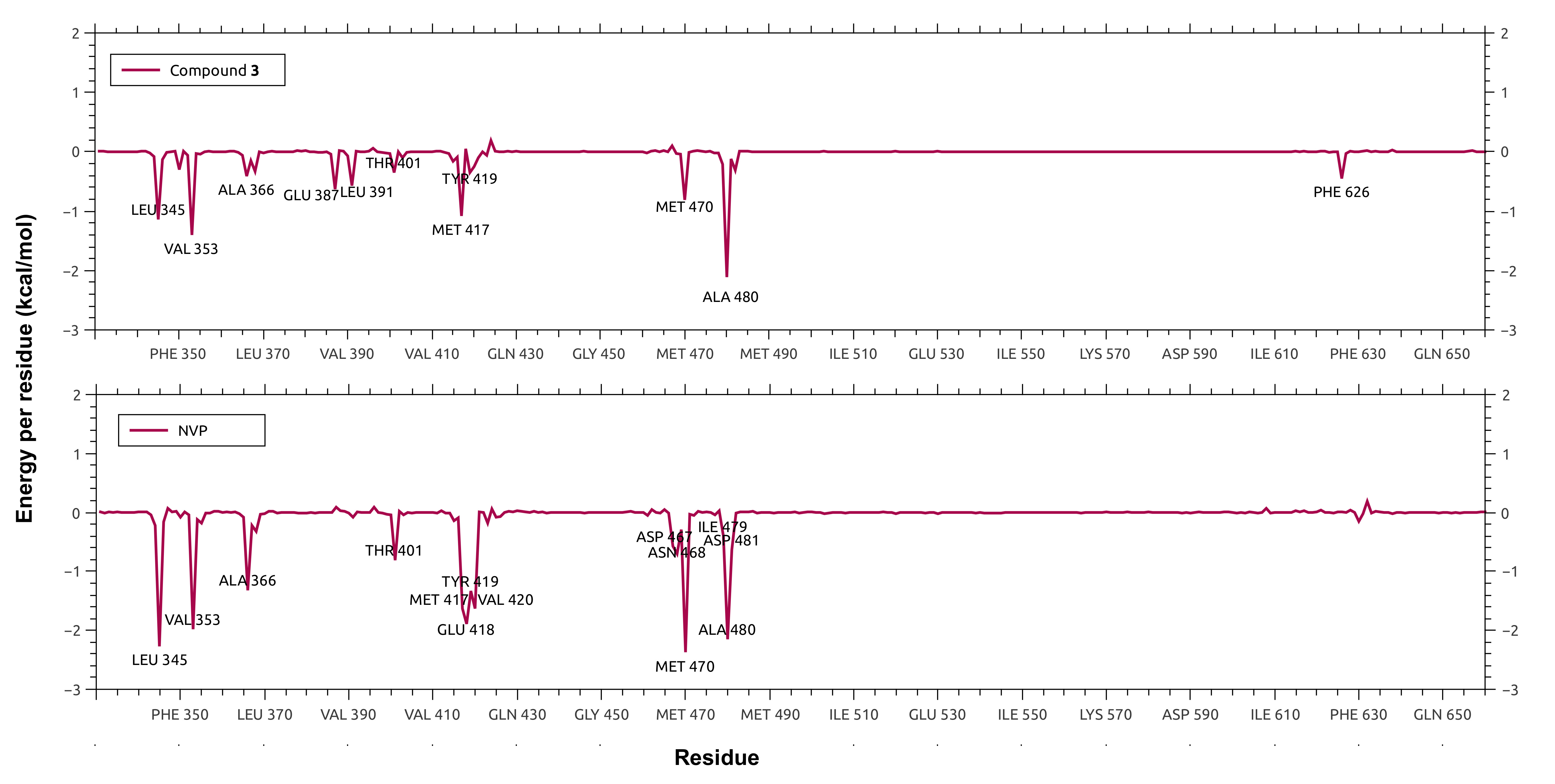
Figure S5** Per residue contributions to the free energy of binding of the complexes PKC- α / compound **3** and PKC- α / NVP.
